# Proteomic profiles among asymptomatic HTLV-1 carriers and HAM/TSP patients in Peru

**DOI:** 10.1186/1742-4690-11-S1-P127

**Published:** 2014-01-07

**Authors:** Morayma Temoche, Jason Rosado, Carolina Alvarez, Daniel Clark, Eduardo Gotuzzo, Michael Talledo

**Affiliations:** 1 Laboratorio de Epidemiología Molecular, Universidad Peruana Cayetano Heredia, Lima, Peru; 2 Instituto de Medicina Tropical Alexander von Humboldt, Universidad Peruana Cayetano Heredia, Lima, Peru; 3 Rega Institute for Medical Research, Katholieke Universiteit Leuven, Leuven, Belgium; 4 Laboratorios de Investigación y Desarrollo, Universidad Peruana Cayetano Heredia, Lima, Peru; 5 Facultad de Medicina, Universidad Peruana Cayetano Heredia, Lima, Peru; 6 Hospital Nacional Cayetano Heredia, Lima, Peru

## 

High proviral loads have been described in HTLV-1-associated myelopathy/tropical spastic paraparesis (HAM/TSP) patients in comparison to asymptomatic HTLV-1 carriers (AC). However, biomarkers related to HAM/TSP progression have not been identified. We analyzed differential proteome changes by two-dimensional gel electrophoresis (2D-electrophoresis) to identify spots of proteins in plasma samples of three groups of patients: five AC, nine HAM/TSP patients: five patients with EDSS scores of 1.0-5.0 (=mild HAM/TSP) and 4 patients with EDSS scores of 5.5-9.0 (=severe HAM/TSP). Proteins were extracted from pooled plasma samples from each group of patients. Protein separations were performed by electrofocusing with 11 cm strips at 3-10 pH range, electrophoresis with 12% poliacrilamide gel electrophoresis and silver staining. Analyses were performed in Progenesis Same Spot software; ANOVA was used to compare the profiles of the groups. Six spots of proteins were differentially expressed among these groups: five proteins increased their expression according to HAM/TSP disease progression (highest fold change= 6.1, p=1.671 x10-4; lowest fold change= 1.5, p= 0.014) while one protein was highly expressed in AC and decreased according to HAM/TSP progression (fold change= 5.8, p= 0.002). Further studies to confirm the expression of these proteins in a larger set of samples are still in progress, the identification of these proteins will be performed by mass spectrometry. These results might be promissory to identify biomarkers for HAM/TSP progression.

